# Susceptibility of perivenous macrophages to PRRSV-1 subtype 1 LV and PRRSV-1 subtype 3 Lena using a new vein explant model

**DOI:** 10.3389/fcimb.2023.1223530

**Published:** 2023-07-24

**Authors:** Shaojie Han, Dayoung Oh, Jiexiong Xie, Hans J. Nauwynck

**Affiliations:** Laboratory of Virology, Department of Translational Physiology, Infectiology and Public Health, Faculty of Veterinary Medicine, Ghent University, Merelbeke, Belgium

**Keywords:** porcine reproductive and respiratory syndrome virus, perivenous macrophages, vessel explant, vascular pathology, immunofluorescence staining

## Abstract

Vessel pathology such as increased permeability and blue discoloration is frequently observed with highly pathogenic PRRSV strains. However, data concerning the viral replication in the environment of blood vessels are absent. In the present study, *ex vivo* models with swine ear and hind leg vein explants were established to study the interaction of PRRSV-1 subtype 1 reference strain LV and highly pathogenic subtype 3 strain Lena with perivenous macrophages. The replication characteristics of these two strains were compared in vein explants by immunofluorescence analysis. The explants maintained a good viability during 48 hours of *in vitro* culture. We found that CD163-positive macrophages were mainly present around the veins and their number gradually decreased with increasing distance from the veins and longer incubation time. More CD163^+^Sn^-^ cells than CD163^+^Sn^+^ cells (6.6 times more) were observed in the vein explants. The Lena strain demonstrated a higher replication level than the LV strain, with approximately 1.4-fold more infected cells in the surrounding areas of the ear vein and 1.1-fold more infected cells in the leg vein explants at 48 hours post inoculation. In both LV and Lena inoculated vein explants, most infected cells were identified as CD163^+^Sn^+^ (> 94%). In this study, an *ex vivo* vein model was successfully established, and our findings will contribute to a better understanding of the vein pathology during viral infections (e.g., PRRS, classical and African swine fever).

## Introduction

Porcine reproductive and respiratory syndrome virus (PRRSV) is a member of the genus *Porarterivirus*, family *Arteriviridae*, order *Nidovirales* (Snijder and Meulenberg, 1998; Nieuwenhuis et al., 2012; Siddell et al., 2019), which was classified into two species, PRRSV-1 (European type) and PRRSV-2 (North American type). These two species display about 40% nucleotide differences (Darwich et al., 2011). Subtype 1 Lelystad virus (LV) and subtype 3 Lena are the two most commonly studied strains of PRRSV-1 (Wensvoort et al., 1991; Karniychuk et al., 2013; Frydas and Nauwynck, 2016; Oh et al., 2020). Macrophages are distributed throughout the whole body, with high concentrations at sites of contact with the external environment, such as respiratory and intestinal tract, in endometrium and fetal placenta, and in lymphoid tissues, which are key players in orchestrating the host immunity. Due to the expression of specific receptors, PRRSV has a limited cell tropism for cells of the macrophage lineage (Duan et al., 1997). Porcine Siglecs [Siglec-1 (also known as sialoadhesin, CD169 or Sn) and Siglec-10] have been shown to be important in viral attachment to the macrophages and internalization (Van Breedam et al., 2010a; Van Breedam et al., 2010b; Van Gorp et al., 2010; Xie et al., 2018). Porcine CD163 was considered as necessary receptor for PRRSV(Burkard et al., 2018), which is not involved in PRRSV binding but mediates the disassembly of the virus (Van Gorp et al., 2010).

Clinical signs of PRRSV-1 infected pigs are very variable, depending on the age, pig breed and virus strain (Frydas et al., 2015; Frydas and Nauwynck, 2016). The location of susceptible cells and the presence of particular receptors can help to explain the pathogenic processes generated by PRRSV. Various investigations have demonstrated that some PRRSV strains can infect not only sialoadhesin-positive cells but also sialoadhesin-negative cells. (Frydas et al., 2015; Frydas and Nauwynck, 2016; Oh et al., 2020). In pregnant sows, one of the classical symptoms caused by PRRSV is reproductive failure, which includes late abortions and early farrowing. At birth, piglets may be weak or dead (stillbirth). Aborted fetuses and stillborn piglets may show starting mummification. The virus’s ability to enter the placenta may be due to an influx of CD163^+^Sn^+^ macrophages into the endometrium and microchimerism in late gestation. (Karniychuk and Nauwynck, 2009; Karniychuk et al., 2012a; Karniychuk et al., 2012b; Karniychuk et al., 2013). In young pigs, single infections with LV-like strains do not cause disease. However, highly pathogenic PRRSV-1 strains, such as the subtype 3 Lena, lead to high fever, anorexia, respiratory problems, lameness and shivering, skin cyanosis and discoloration of ears (Li et al., 2016). The porcine alveolar macrophage (PAM) is considered the primary target cell for PRRSV infection in the respiratory tract, owing to the high expression of both CD163 and Sn receptors. Pulmonary interstitial and intravascular lung macrophages are also susceptible to PRRSV and are an important source of cell-free virus in the blood (Thanawongnuwech et al., 2000). Recently, a new macrophage subset, designated ‘nasal surface macrophages’ (CD163^+^Sn^-^), was identified in the nose, which was also susceptible to many PRRSV strains *in vitro* (Oh et al., 2020).

PRRS was originally called blue ear disease, due to the occurrence of blue discoloration of the ears. In addition, cyanosis and massive exudate in the abdominal and thoracic cavities may be observed, mainly during infections with highly pathogenic strains (Paton et al., 1991; Karniychuk et al., 2010). PRRSV infections often cause vascular damage (Scruggs and Sorden, 2001) and during an *in vivo* experiment, PRRSV (HuN4) was detected in the environment of small blood vessels in the tonsils and lymph nodes, and highly pathogenic PRRSV (HP-PRRSV) positive cells were found in veins surrounding the brain (Hu et al., 2013). Han et al. described severe lung lesions caused by HP-PRRSV infection due to extensive hemorrhages and infiltration of immune cells in the vascular systems (Han et al., 2014) and Sun et al. reported that a PRRSV infection disrupts the function of endothelial cells, leading to permeability changes of the lung vascular system (Sun et al., 2022). A recent study reported that brain resident macrophages located in the perivascular space play a significant role in phagocytosis and pathophysiological functions (Faraco et al., 2017). These findings suggest that PRRSV may be able to replicate in the vein-associated macrophages and trigger the development of clinical symptoms (hemorrhage, viremia and discoloration) in pigs.

The interaction between PRRSV and vascular macrophages underlying this vascular pathology has never been investigated. Hence, the main purpose of this study was to develop an *ex vivo* vessel culture system, to examine if macrophages are present in the surrounding of veins and if so, to characterize the perivenous macrophages (presence of PRRSV receptors sialoadhesin and CD163) and to examine their susceptibility to two PRRSV-1 strains, LV and Lena.

## Materials and methods

### Animals and collection of veins

Three 3-week-old conventional pigs from a PRRSV negative farm were used. The pigs were euthanized with 12.5mg/kg body weight pentobarbital (Kela, Hoogstraten, Belgium). After exsanguination, the ears and hind legs of the pigs were cut off from the carcass. Incisions were made using sterile tweezers and a blade until the veins were exposed. Auricular and saphenous veins were removed from the surrounding tissues, and then immediately placed in transport medium containing phosphate buffer saline (PBS), supplemented with 0.1 mg/mL gentamicin (Invitrogen, Gent, Belgium), 0.1 mg/mL streptomycin (Certa, Eigenbrakel, Belgium), and 100 U/mL penicillin (Continental Pharma, Puurs, Belgium). Afterwards, the veins were cut into small pieces (0.5 cm), placed on a fine-meshed gauze (Glorieux et al., 2007) in a 24-well plate and cultured for 48 h (37°C, 5% CO_2_) in complete RPMI 1640 medium containing 10% Fetal Bovine Serum (FBS) (Sigma-Aldrich, USA), 1 mM sodium pyruvate (Gibco, USA), 1% non-essential amino acids (Gibco, USA), 0.05 mg/mL gentamycin (Gibco, USA), 0.1 mg/mL streptomycin (Gibco, USA), and 100 U/mL penicillin (Gibco).

### Evaluation of tissue viability in veins explants

To evaluate the viability of the explants, an *In Situ* Cell Death Detection Kit was used (Roche Diagnostics, Switzerland) based on terminal deoxynucleotidyl transferase dUTP nick end-labeling (TUNEL). Samples were collected in methylcellulose and frozen at 0, 12, 24, and 48 h post-culture, 9 µm cryosections were made and TUNEL stained according the manufacturer’s instruction. The sections were analyzed using a confocal microscope (Leica Microsystems GmbH, Heidelberg, Germany). The percentage of TUNEL-positive cells was determined within the endothelium, smooth muscle cell layer, and connective tissue in five randomly selected fields with a 10x ocular lens and a 40x objective.

### Viral inoculation of the explants

Two PRRSV strains were used in this study: LV (prototype PRRSV-1, subtype 1; 13th passage in porcine alveolar macrophages (PAM); 10^6.3^ TCID_50_/ml) and Lena (prototype PRRSV-1, subtype 3; 4th passage in PAM; titer 10^6.3^ TCID_50_/ml). After 1 h preincubation of the explants in complete medium at 37°C in the presence of 5% CO_2_, explants were inoculated with 500 µl of each virus per well (containing 10^6.0^ TCID50 virus). Explants that were incubated with 500 μL of medium were used as mock controls. After 1 h inoculation, another 500 μL of complete medium were added to each well. Explants were collected at 0, 12, 24 and 48 hours post-inoculation (hpi), washed three times with transport medium and immediately embedded in methylcellulose medium (Thermo Fisher GmbH, Kandel, Germany) and stored at −70°C.

### Analysis of the perivenous macrophage distribution and infection by immunofluorescence staining and confocal microscopy

The methylcellulose-embedded frozen tissue samples were cryosectioned at a 9 μm thickness using a cryostat at −20°C and loaded onto 3-aminopropyltriethoxysilane-coated (Sigma-Aldrich, St. Louis, MO, USA) glass slides. Tissue sections were then fixed and permeabilized in 100% methanol for 15 min at -20°C. Afterwards, the sections were washed in PBS. To identify the endothelial cells and smooth muscle cells in the ear and leg veins, the sections were incubated in a polyclonal rabbit antibodies against human von Willebrand factor (vWF, IgG, 1:50, Agilent) together with a monoclonal antibody (mAb) against human smooth muscle cells (SMC, clone 1A4, IgG2a, 1:50, Agilent) for 1h at 37°C, followed by an incubation with goat anti-rabbit IgG Texas red (1:100, Invitrogen) and goat anti-mouse IgG2a Alexa fluor 488 (1:200, Invitrogen).

To identify the different macrophage subpopulations in the vein explant, a double IF staining against CD163 and Sn was performed. Sections were incubated with a mouse mAb against porcine CD163 (clone 2A10/11, IgG1, Bio-Rad, Oxford, UK, 1:1800), a mouse anti-human Sn mAb (clone 26B2, lgG2b, in house, undiluted supernatant) (De Schryver et al., 2016) (Van Breedam et al., 2011) for 1 h at 37°C, followed by three washings with PBS and incubation with secondary antibodies: goat anti-mouse IgG1 Alexa fluor 647 (1:200, Invitrogen) and goat anti-mouse IgG2b Alexa fluor 488 (1:30, Invitrogen) for 1 h at 37°C. A mouse anti-pseudorabies virus gD mAb, clone 13D12 (IgG1) (Nauwynck and Pensaert, 1995) and a mouse anti-PCV2 capsid mAb, clone 12E12 (IgG2b) (Saha et al., 2012) were used as isotype controls. Non-specific binding sites were blocked with 10% of goat serum. Cell nuclei were counterstained with Hoechst 33342 (10 μg/mL, Invitrogen) for 10 min at 37°C.

**Figure 1:**
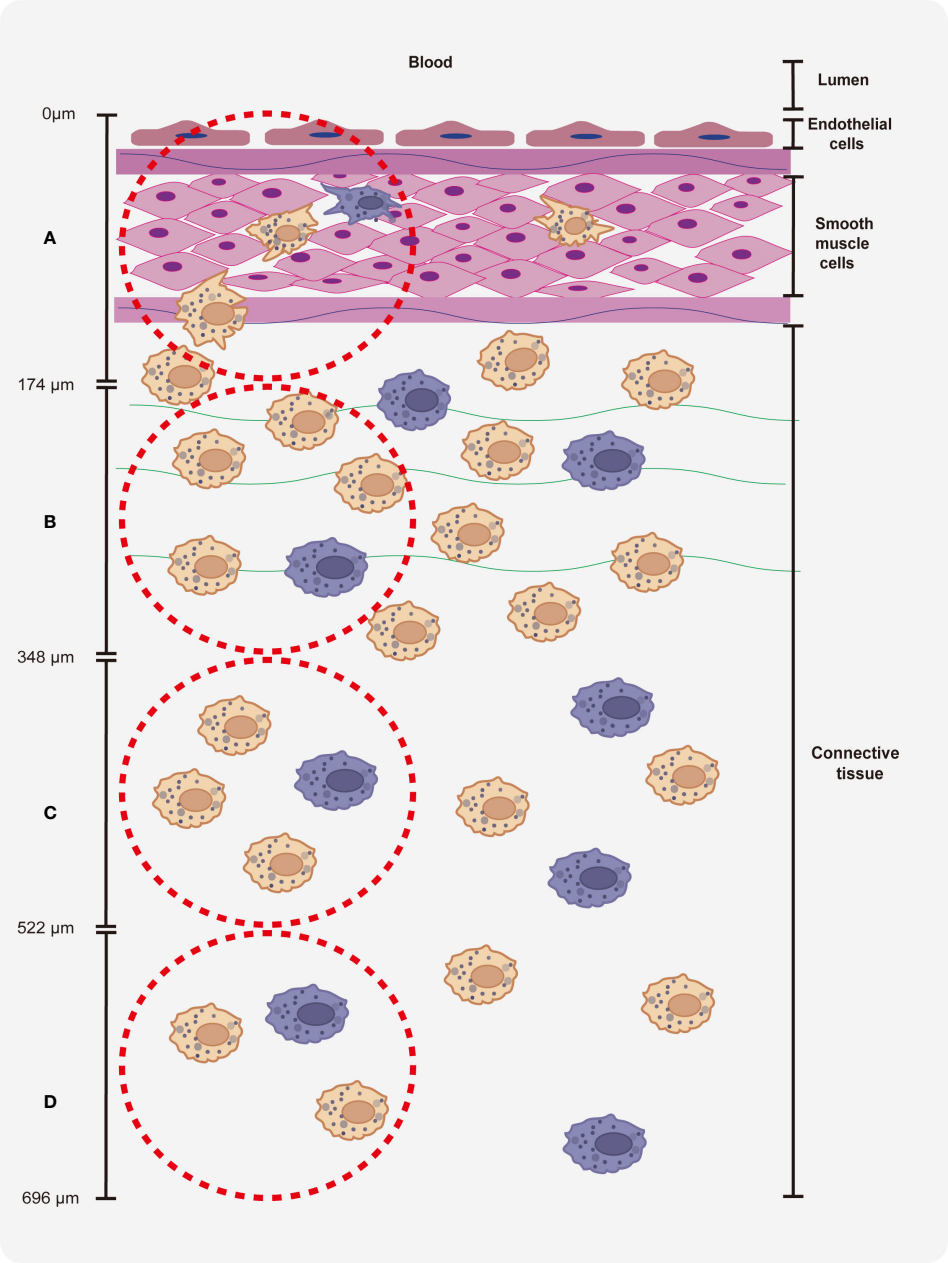
Quantitation of perivenous macrophages in four different ROIs (red frame). Each ROI was observed by confocal microscopy (10x ocular lens and 63x objective): area **(A)** (0 - 174 µm), area **(B)** (174 - 348 µm), area **(C)** (348 - 522 µm) and area **(D)** (522 - 696 µm), starting from the endothelial cell layer moving further into the surrounding connective tissue.

To identify the macrophages in different parts of the vein explants (**Figure 1**), we made four regions of interest (ROI), which starts from the lumen side of the vessel to the surrounding connective tissue. Region A covers endothelial cell layers and smooth muscle cells of the vein, region B covers smooth muscle cells and cells from connective tissue, region C and region D covers cells from connective tissue. For the quantitation and characterization of the perivenous macrophages in each ROI (area of 0.03 mm^2^ (30276 μm^2^)), a total number of around 100 cells in each ROI was analyzed.

In order to analyze the susceptibility of perivenous macrophages to PRRSV, a triple IF staining against CD163, Sn and PRRSV was performed. Sections were incubated with a mouse monoclonal antibody (mAb) against porcine CD163 (clone 2A10/11, IgG1, Bio-Rad, Oxford, UK, 1:1800), a mouse anti-human Sn mAb (clone 26B2, lgG2b, in house, undiluted supernatant)(De Schryver et al., 2016), and an anti-PRRSV nucleocapsid (N) mAb (13E2, IgG2a, in house, 1:50 dilution)(Van Breedam et al., 2011) for 1 h at 37°C, followed by three washings with PBS and incubation with secondary antibodies: goat anti-mouse IgG1 Alexa fluor 647 (1:200, Invitrogen), goat anti-mouse IgG2b Alexa fluor 488 (1:30, Invitrogen), and goat anti-mouse IgG2a Alexa Fluor 594 (1:200, Invitrogen) for 1 h at 37°C. A mouse anti-pseudorabies virus gD mAb, clone 13D12 (IgG1) (Nauwynck and Pensaert, 1995), a mouse anti-pseudorabies virus gB mAb, clone 1C11 (IgG2a) (Nauwynck and Pensaert, 1995) and a mouse anti-PCV2 capsid mAb, clone 12E12 (IgG2b) (Saha et al., 2012) were used as isotype controls. Non-specific binding sites were blocked with 10% goat serum. Cell nuclei were counterstained with Hoechst 33342 (10 μg/mL, Invitrogen) for 10 min at 37°C. The viral antigen-positive cells were quantified in perivenous macrophages in the entire cryosection, and then classified based on the expression of CD163 and Sn.

To check PRRSV antigen-positive cells for apoptosis, a terminal deoxynucleotidyl transferase dUTP nick end labeling (TUNEL) assay was combined with immunofluorescent staining for PRRSV nucleocapsid. The TUNEL and viral antigen double positive cells were counted in the section. The percentage of TUNEL^+^PRRSV^+^ cells were calculated within the population of PRRSV infected macrophages.

### Statistical analysis

All analyses were completed with GraphPad Prism statistical software package version 9.0 (GraphPad, San Diego, CA, USA). Differences between sample groups were analyzed using Two-way analysis of variance (ANOVA) followed by Tukey’s *post hoc* test. All data were presented as mean ± standard deviation (SD) from three different pigs. Results with a *p*-value of < 0.05 were considered significantly different.

## Results

### Structure of the ear and leg vein explants

**Figure 2:**
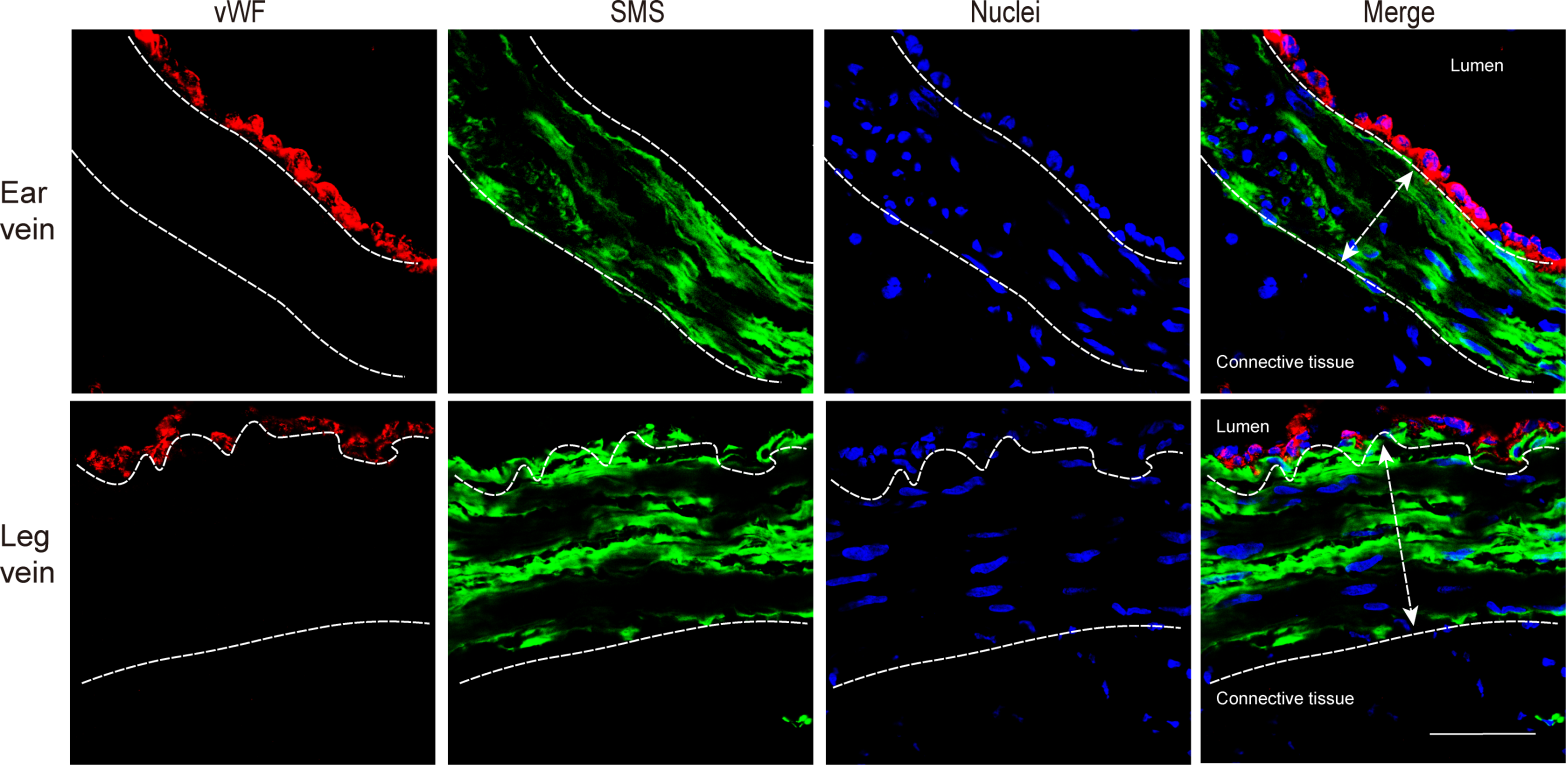
Immunofluorescence staining of endothelial cells (red) and smooth muscle cells (green) in ear vein and leg vein explants. The thickness of smooth muscle cells was analyzed by confocal microscopy (10x ocular lens and 63x objective). The average thickness of the smooth muscle cell layer of the ear vein was 65.8 µm (white double headed arrow), and the average thickness of the smooth muscle cell layer of the leg vein was 102 µm (white double headed arrow). Scale bar: 50 μm.

The structure of vein explants was examined by a double immunofluorescence staining (**Figure 2**). Both ear and leg vein explants consisted of three different layers, including an innermost layer of endothelial cells (red), a middle layer of smooth muscle cells (green) and an adventitia layer (connective tissue with various cell types).

### Evaluation of explant viability of ear and leg vein explants

**Figure 3:**
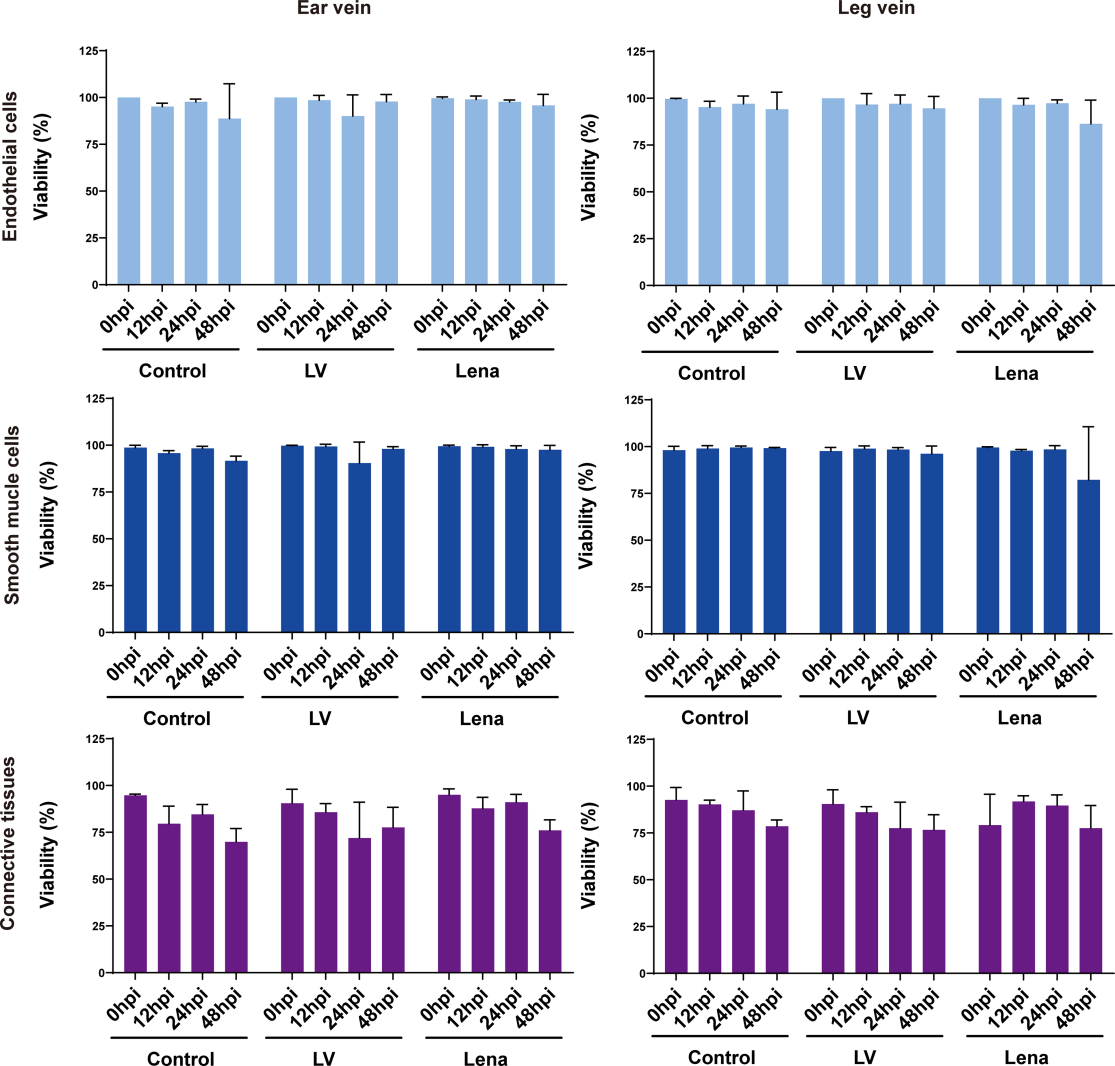
Evaluation of the effect of *in vitro* cultivation and PRRSV inoculation on the viability of ear and leg vein explants as assessed by a TUNEL staining at different hours of cultivation. TUNEL-positive cells were counted within the endothelium, smooth muscle cells and connective tissue after random selection of five consecutive fields. All data are expressed as mean value of three animals ± SD.

To evaluate the effect of *in vitro* cultivation and PRRSV inoculation on the viability of vein explants, the percentage of TUNEL positive cells was calculated at 0, 12, 24, and 48 h after mock and PRRSV inoculations (**Figure 3**; **Supplementary Figures 1**, **2**).

In ear vein explants at 48 hpi, the cell viability of endothelial cells, smooth muscle cells, and connective tissue decreased to 88.7 ± 18.7%, 91.7 ± 2.5%, and 69.9 ± 7.1%, respectively, in mock inoculated explants; 97.8 ± 3.9%, 98.0 ± 1.3%, and 77.6 ± 10.7%, in LV-inoculated explants; and 95.8 ± 6.0%, 97.5 ± 2.3%, and 76.1 ± 5.6% in Lena-inoculated explants.

In leg vein explants at 48 hpi, the cell viability of endothelial cells, smooth muscle cells, and connective tissue decreased to 94.1 ± 9.1%, 99.1 ± 0.3%, and 78.6% ± 3.4%, respectively, at 48 hpi in mock inoculated explants, 94.6 ± 6.4%, 96.1 ± 4.1%, and 76.7 ± 8.0% in LV-inoculated explants, and 86.3 ± 12.7%, 82.2 ± 28.4%, and 77.5 ± 12.1% in Lena-inoculated explants.

Overall, more than 70% of the cells in ear/leg vein explants remained viable within 48 hours of *in vitro* culture, and PRRSV inoculation did not affect viability as determined by the evaluation of cell viability in endothelial cells, smooth muscle cells, and connective tissue.

### Quantitation of CD163^+^Sn^+^ and CD163^+^Sn^-^ cells in vein explants

**Figure 4:**
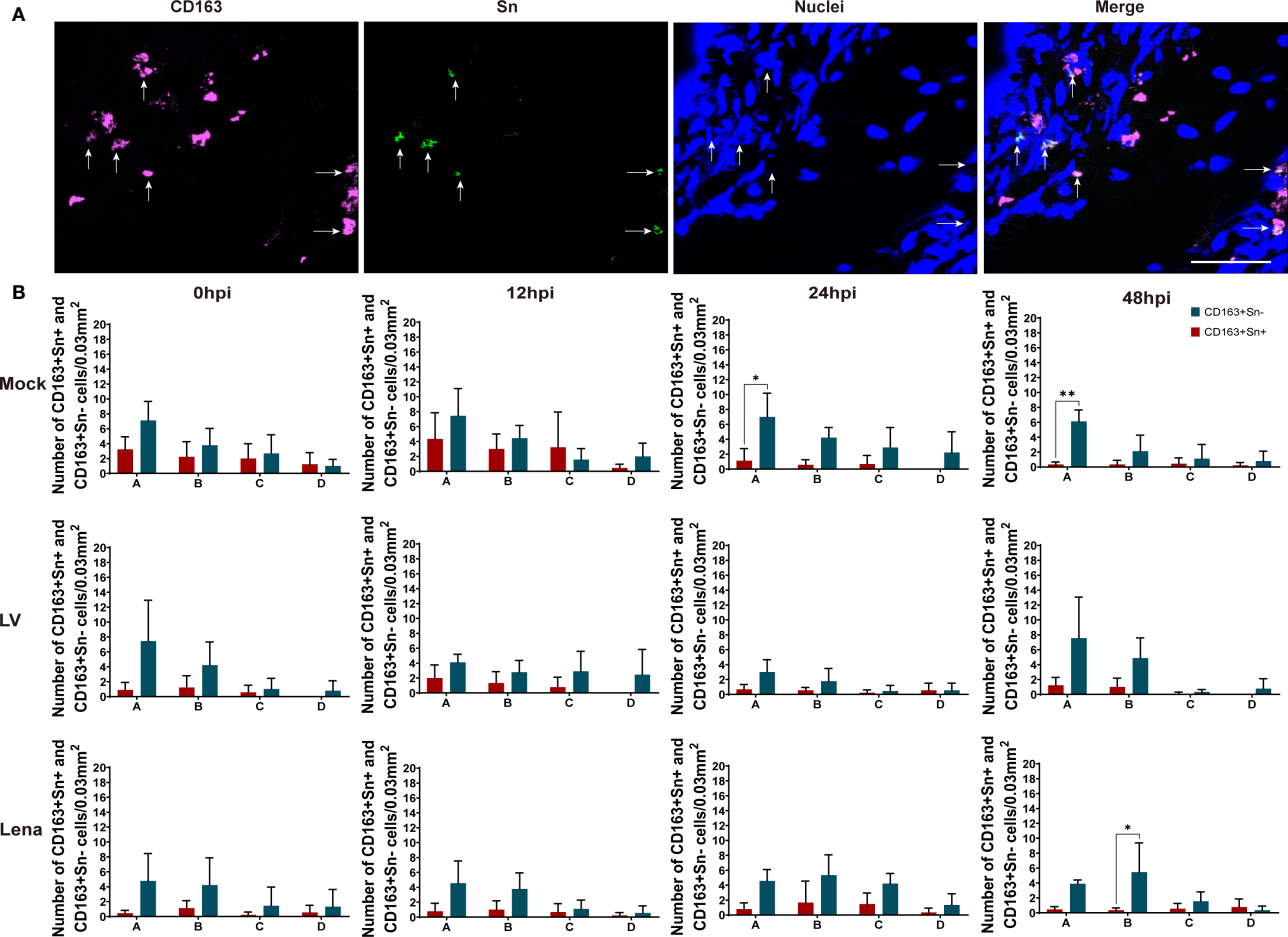
Quantitation and distribution of CD163^+^Sn^-^ and CD163^+^Sn^+^ perivenous macrophages in mock-inoculated and PRRSV (LV and Lena)-inoculated ear vein explants. **(A)** Cryosections of the vein explant were immunostained for CD163 (magenta) and Sn (green) with a double immunofluorescence staining (Picture was taken from Area A). Nuclei were visualized with Hoechst (blue). Scale bar: 50 μm. **(B)** Quantification of CD163^+^Sn^+^ and CD163^+^Sn^-^ perivenous macrophages in areas A (0 - 174 µm), B (174 - 348 µm), C (348 - 522 µm) and D (522 - 696 µm) at different timepoints. Statistical significance was determined by Two-way ANOVA followed by Tukey’s multiple comparison *post hoc* test (*P < 0.05; **P < 0.01). All data are expressed as mean value of three animals ± SD.

**Figure 5:**
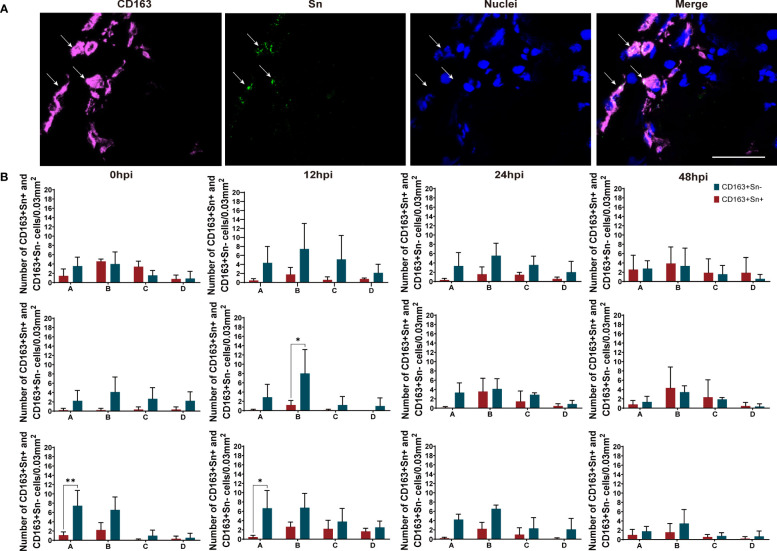
Quantitation and distribution of CD163^+^Sn^+^ and CD163^+^Sn^-^ perivenous macrophages in mock-inoculated and PRRSV (LV and Lena)-inoculated leg vein explants. **(A)** Cryosections of the vein explant were immunostained for CD163 (magenta) and Sn (green) with a double immunofluorescence staining (Picture was taken from Area B). Nuclei were visualized with Hoechst (blue). Scale bar: 50 μm. **(B)** Quantification of CD163^+^Sn^+^ and CD163^+^Sn^-^ perivenous macrophages in areas A (0 - 174 µm), B (174 - 348 µm), C (348 - 522 µm) and D (522 - 696 µm) at different timepoints. Statistical significance was determined by Two-way ANOVA followed by Tukey’s multiple comparison *post hoc* test (*P < 0.05). All data are expressed as mean value of three animals ± SD.

As shown in **Figures 4** and **5**, two different subsets of perivenous macrophages were identified in ear and leg vein explants by double immunofluorescence staining: CD163^+^Sn^+^ and CD163^+^Sn^-^ cells. To quantitate the CD163^+^Sn^+^ and CD163^+^Sn^-^ cells in the explants, four different ROIs were identified: area A (0 - 174 µm), area B (174 - 348 µm), area C (348 - 522 µm) and area D (522 - 696 µm), starting from the endothelial cell layer moving further into the surrounding connective tissues (**Figure 1**, **Supplementary Figures 3**, **4**). CD163^+^ cells were widely distributed in different parts of vein explants during the 48h cultivation period. The highest number of CD163^+^ cells was present in area A (0 - 174 µm) of the ear vein explant (approximately 4 - 11 cells/0.03 mm^2^) and area B (174 - 348 µm) of the leg vein explant (approximately 4 - 10 cells/0.03 mm^2^) and decreased further away from the vein. More CD163^+^Sn^-^ cells than CD163^+^Sn^+^ were observed in the ear vein explants (6.4 times in area A, *P* = 0.03) and leg vein explants (6.6 time in area B, *P* = 0.03).

### Quantitation and characterization of viral antigen positive cells in vein explants

**Figure 6:**
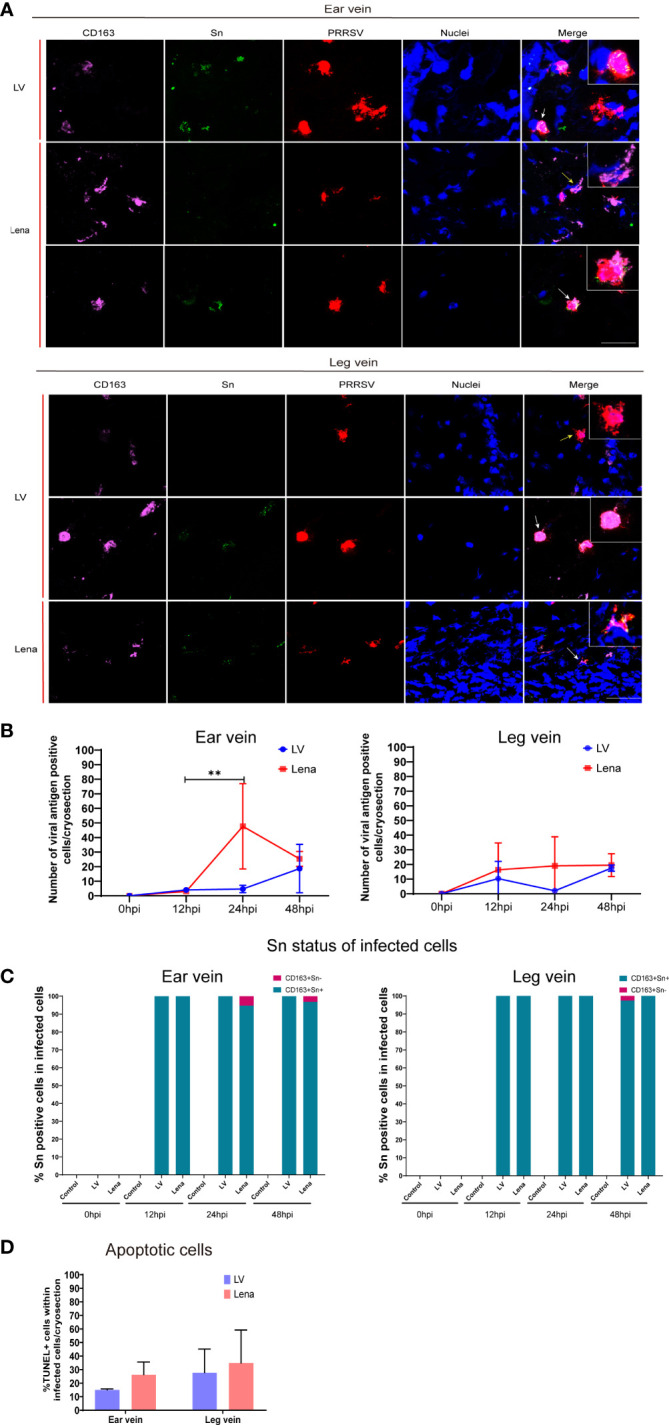
PRRSV replication kinetics in the perivenous macrophages in the vein explants at different hours post-inoculation (0, 12, 24, and 48 hpi). **(A)** Representative pictures of viral antigen positive CD163^+^Sn^+^ (white arrow) and CD163^+^Sn^-^ (yellow arrow) cells in ear and leg vein explants by a triple immunofluorescence staining, all viral antigen positive cells were observed in the connective tissues of vein explant; nuclei are visualized with Hoechst staining (blue). Scale bar: 50 μm. **(B)** Quantitation of PRRSV positive perivenous macrophages in ear and leg vein explants. **(C)** Sn status of infected cells (CD163^+^) at different time points. **(D)** TUNEL-positive cells (apoptotic cells) were quantitated in PRRSV-positive cells in ear vein and leg vein explants at 48hpi. Statistical significance was determined by Two-way ANOVA followed by Tukey’s multiple comparison *post hoc* test (**P < 0.01). All data are expressed as mean value of three experiments ± SD.

The number of viral antigen positive cells and their identity was determined by a triple immunofluorescence staining for CD163, Sn and PRRSV nucleocapsid of cryosections from ear and leg vein explants, collected at 0, 12, 24, and 48 hpi (**Figure 6A**; **Supplementary Figure 5**). The viral antigen positive cells were assessed at different time points in the LV and Lena inoculated groups (**Figure 6B**).

At 48 hpi, more PRRSV positive cells were found in the Lena-inoculated group than LV-inoculated group in the ear vein explant (Lena: 25.3 ± 5.1 cells/cryosection, LV: 18.6 ± 16 cells/cryosection) and leg vein explant (Lena: 19.5 ± 7.7 cells/cryosection, LV: 17.5 ± 2.1 cells/cryosection) (**Figure 6B**). CD163^+^Sn^+^ cells and CD163^+^Sn^-^ cells showed a different susceptibility to PRRSV (**Figure 6C**). Most PRRSV-infected cells were CD163^+^Sn^+^ cells. Exceptionally, a few infected CD163^+^Sn^-^ cells were found the in LV-inoculated and Lena-inoculated explants (6.8% of the infected cells were CD163^+^Sn^-^ in the Lena-inoculated ear vein explants and 1.8% in the LV-inoculated leg vein explants) (**Figure 6C**). Furthermore, more apoptotic cells were observed in Lena infected cells than in LV infected cells (26.23 ± 9.3% versus 14.99 ± 0.8% in ear vein explant and 34.81 ± 24.4% versus 27.69 ± 17.4% in leg vein explant) (**Figure 6D**). However, no statistical difference was observed between the Lena inoculated group and the LV inoculated group.

## Discussion

Macrophages are distributed throughout the body and may be classified into multiple subtypes based on the expression of different receptors, with different subpopulations of macrophages displaying variable degrees of sensitivity to infections. The interaction between PRRSV and macrophages is mainly determined by the expression of binding and internalization receptors, such as Siglec-1 (also known as Sn and CD169) and Siglec-10 and the disassembly mediator CD163 (Van Breedam et al., 2010a; Xie et al., 2018). Due to the high expression of CD163 and Sn, alveolar macrophages, lung interstitial and intravascular macrophages, lymphoid macrophages and endometrial and placental macrophages are important target cells for PRRSV (Karniychuk and Nauwynck, 2009; Gómez-Laguna et al., 2013). In lymphoid tissues, also Siglec-10 and CD163 double positive cells become infected. Previous studies from our laboratory with nasal explants found that CD163^+^Sn^+^ and CD163^+^Sn^-^ macrophages have different susceptibilities for PRRSV-1 subtype 1 LV and PRRSV-1 subtype 3 Lena (Frydas et al., 2013). Up till now, it is not clear which alternative binding and internalization receptor is used in CD163^+^Sn^-^ nasal macrophages. In a recent review, it has been reported that perivascular macrophages have multiple functions in the microenvironment of different organs, such as regulation of blood vessel permeability, elimination of potential pathogens, antigen presentation and immune regulation in the brain (Lapenna et al., 2018). Extensive research has been conducted on perivascular macrophages in the central nervous system, highlighting their critical role in maintaining tissue homeostasis and their involvement in various pathological processes (Zheng et al., 2022). The utilization of *ex vivo* tissues provides the advantage of real-time visualization, enabling the observation of the dynamic progression of certain pathogenic processes (Grivel and Margolis, 2009). This is the first study on the interaction of PRRSV with perivenous macrophages in a vein explant model. It forms a good model for future studies of other viruses associated with vascular pathology, such as CSFV, or even Ebola virus in humans.

In this study, we established a novel vein explant culture system to study PRRSV interaction with vascular macrophages. We first evaluated the viability of explants at different parts of the explant, including endothelial cell layer, smooth muscle cell layer and connective tissue (**Supplementary Figures 1**, **2**). In both the ear vein and leg vein explants, the viability only slightly decreased over 48 hours *in vitro* culture. Based on our *in vitro* culture system, the cell viability of the explants can be maintained at 70% even after 48 hours of culture. This new vein explant model will help us to further study the role of vascular macrophages in vasculopathy (increased permeability, hemorrhage, viremia and discoloration) caused by PRRSV and other vasculopathy-inducing viruses such as classical swine fever virus and African swine fever virus in cellular pathogenesis.

Based on the expression of CD163 and Sn, two different subsets of perivenous macrophages (CD163^+^Sn^+^ and CD163^+^Sn^-^ cells) were observed in ear and leg veins by a double immunofluorescence staining. The perivascular CD163^+^ macrophages are widely distributed in the vein explant and their number decreases over distance from the endothelial cell layer of the vessel. Likewise, in a human study, CD163^+^ cells were also observed in the perivascular area of normal skin (Chong et al., 2015). Most CD163^+^ macrophages were located in area A of ear vein explants (approximately 4-11 cells/0.03 mm^2^) and area B of leg explants (approximately 4 - 10 cells/0.03 mm^2^). In summary, the majority of CD163^+^ cells are predominantly located in the perivascular space close to the vessel (0 - 348 μm), suggesting that it may be involved in the maintenance of different vascular functions. Previous studies in mice have demonstrated that Sn^+^ cells are distributed in the perivascular region of the renal interstitium and participate in vascular homeostasis (Chinnery et al., 2010; Karasawa et al., 2015). In our work, Sn^+^ cells were also observed in the perivascular areas of ear vein and leg vein explants; however, most venous macrophages were CD163^+^Sn^-^ cells, suggesting that Sn is most probably not playing a key role in functional processes of the ear or leg vessels in pig.

Highly pathogenic PRRSV strains affect the pulmonary vascular system and can exacerbate respiratory-related disease, increasing host mortality and morbidity (Han et al., 2014). The role of porcine CD163^+^ perivascular macrophages in a PRRSV infection has not been studied yet. Therefore, the potential for PRRSV-1 subtype 1 LV and PRRSV-1 subtype 3 Lena infection of perivenous macrophages was investigated in the present study. The number of viral antigen-positive cells increased over incubation time (**Figure 6B**), indicating that PRRSV can successfully replicate in our vein explant model. In both vein explant systems, the number of Lena-infected cells was greater than the number of LV-infected cells. Similar results were observed in nasal mucosal explants where a higher number of viral antigen-positive cells was identified in the highly pathogenic Lena-inoculated group (Frydas et al., 2013). In addition, higher numbers of infected cells were observed in ear vein explants than in leg vein explants, which may explain why blue discoloration was mainly observed on ears in the field. The latter was the basis for the name ‘blue ear disease’, given to PRRS in the early nineties. Notably, the majority of infected cells were CD163^+^Sn^+^ in both LV and Lena-inoculated group; only a few cells were CD163^+^Sn^-^ (6.8% of the infected cells in the Lena-inoculated ear vein explants and 1.8% in the LV-inoculated leg vein explants). This is in contrast with the CD163^+^Sn^-^ nasal macrophages in the lamina propria of the nasal mucosa that can also be infected by PRRSV (Frydas and Nauwynck, 2016; Oh et al., 2020). Taken together, our results suggest that Sn is playing an important role as binding/internalization receptor for PRRSV in perivenous macrophages. Upregulation of Sn in this subpopulation of macrophages may activate PRRSV replication. IL-10 and corticosteroids are known to stimulate Sn expression (Delputte et al., 2007; Singleton et al., 2018). Future work will be done to examine if these molecules may increase Sn expression and subsequently PRRSV replication in the perivascular region and to analyze the consequence on the integrity of the endothelial cell layer and the tonus of the smooth muscles.

Apoptosis plays an important role in the immune system to protect the host from pathogenic microorganisms (Orzalli and Kagan, 2017). Many studies have demonstrated that PRRSV infected macrophages undergo apoptosis (Lee and Kleiboeker, 2007; Costers et al., 2008; Karniychuk et al., 2011) and that apoptosis induced by PRRSV is virulence-dependent (Sánchez-Carvajal et al., 2021). In agreement with previous studies, we found that PRRSV infected perivenous macrophages go into apoptosis and that more apoptotic cells were present in Lena infected macrophages than in LV infected macrophages in the vein explants.

Additional work will be performed to understand the specific role of perivascular macrophages and the effect of a PRRSV infection. Very interesting is the role of certain cytokines that might be released from PRRSV infected perivenous macrophages and their impact on the smooth muscle cell (vasoconstriction/vasodilation) and permeability of the endothelial cell layer. In this context, single cell transcriptomics will be performed in the future.

Filoviruses in humans are also infecting macrophages and cause increased blood vessel permeability, even leading to hemorrhages (Kortepeter et al., 2011). The present work is very interesting in the context of vessel pathology caused by other viruses, such as African swine fever virus (ASFV) and classical swine fever virus (CSFV) in pigs and Ebola virus in humans (Blome et al., 2017; Salguero, 2020). In light of these findings, the vein explant model might be a useful *in vitro* model for elucidating the mechanisms underlying the development of vascular disorders induced by pathogenic microorganisms. In the future, it will be examined how PRRSV and other porcine vessel-pathology-inducing viruses with a tropism for macrophages are affecting the blood vessel permeability. Therefore, the pathogenesis studies that will be performed with the present *in vitro* animal vein model will be very useful in elucidating the underlying mechanism of viral induced pathologies with viruses that have a tropism for macrophages in animals and humans.

In conclusion, this study demonstrates for the first time the distribution and characterization of porcine perivenous macrophages and their susceptibility to PRRSV using a vein explant model. A large number of CD163^+^Sn^-^ and a low number of CD163^+^Sn^+^ perivenous macrophages were distributed around the vessels and mainly CD163^+^Sn^+^ perivenous macrophages were susceptible to PRRSV (Lena > LV). These results are relevant for a better understanding of blood vessel pathology in the pathogenesis of PRRS and other viral diseases.

## Data availability statement

The original contributions presented in the study are included in the article/**Supplementary Material**. Further inquiries can be directed to the corresponding author.

## Ethics statement

The animal study was reviewed and approved by Local Ethical Committee of the Faculty of Veterinary Medicine, Ghent University.

## Author contributions

SH designed and performed all the experiments, analyzed the data and wrote the manuscript, HN and DO conceived and designed the study and helped in writing the manuscript. JX helped in writing the manuscript. All authors read and approved the final manuscript.

## Supplementary material

Supplementary Figure 1 Evaluation of the cell viability in ear vein explants by TUNEL assay. TUNEL positive cells were quantitated in endothelial cell layers (En), smooth muscles cells (SMS) and connective tissues (CT) in the mock-inoculated group, LV-inoculated group and Lena-inoculated group at different time points. Scale bar: 50 μm.

Supplementary Figure 2 Evaluation of cell viability in leg vein explants by TUNEL assay. TUNEL positive cells were quantitated in endothelial cell layers (En), smooth muscles cells (SMS) and connective tissues (CT) in the mock-inoculated group, LV-inoculated group and Lena-inoculated group at different time points. Scale bar: 50 μm.

Supplementary Figure 3 Distribution of CD163^+^Sn^+^ and CD163^+^Sn^-^ cells in different parts of ear vein explants. Cryosections of the vein explant were immunostained for CD163 (magenta) and Sn (green) with a double immunofluorescence staining. Nuclei were visualized with Hoechst (blue). For the quantitation and characterization of the perivenous macrophages in each ROI (area of 0.03 mm^2^ (30276 μm^2^)), a total number of around 100 cells in each ROI was analyzed. Scale bar: 50 μm.

Supplementary Figure 4 Distribution of CD163^+^Sn^+^ and CD163^+^Sn^-^ cells in different parts of leg vein explants. Cryosections of the vein explant were immunostained for CD163 (magenta) and Sn (green) with a double immunofluorescence staining. Nuclei were visualized with Hoechst (blue). For the quantitation and characterization of the perivenous macrophages in each ROI (area of 0.03 mm^2^ (30276 μm^2^)), a total number of around 100 cells in each ROI was analyzed. Scale bar: 50 μm.

Supplementary Figure 5 Positive control and isotype controls for the CD163/Sn/PRRSV triple immunofluorescence staining. PAMs inoculated with PRRSV were used as positive control for the CD163/Sn/PRRSV triple immunofluorescence staining. A mouse anti-pseudorabies virus gD mAb, clone 13D12 (IgG1) (Nauwynck and Pensaert, 1995), a mouse anti-pseudorabies virus gB mAb, clone 1C11 (IgG2a) (Nauwynck and Pensaert, 1995) and a mouse anti-PCV2 capsid mAb, clone 12E12 (IgG2b) (Saha et al., 2012) were used as isotype controls for the ear and leg vein triple immunofluorescence staining. Scale bar: 25 μm.
