# Supplementary figures and images for: Susceptibility of perivenous macrophages to PRRSV-1 subtype 1 LV and PRRSV-1 subtype 3 Lena using a new vein explant model
Source: Front Cell Infect Microbiol. 2023 Jul 24;13:1223530. doi: 10.3389/fcimb.2023.1223530 (PMC10406384; doi:10.3389/fcimb.2023.1223530)

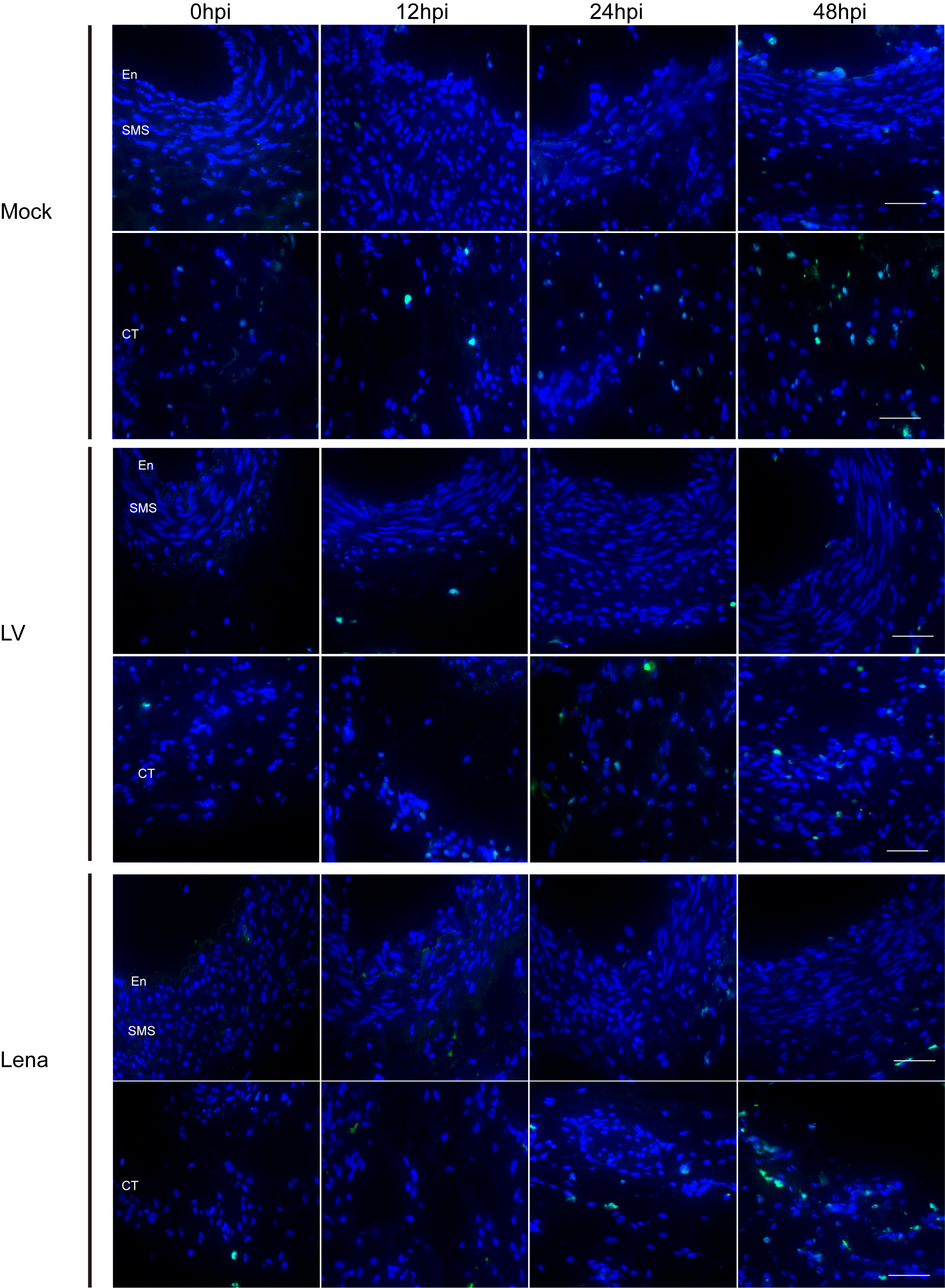

Supplement: Supplementary Figure 1 — Evaluation of the cell viability in ear vein explants by TUNEL assay. TUNEL positive cells were quantitated in endothelial cell layers (En), smooth muscles cells (SMS) and connective tissues (CT) in the mock-inoculated group, LV-inoculated group and Lena-inoculated group at different time points. Scale bar: 50 μm. [file Image_1.tif]

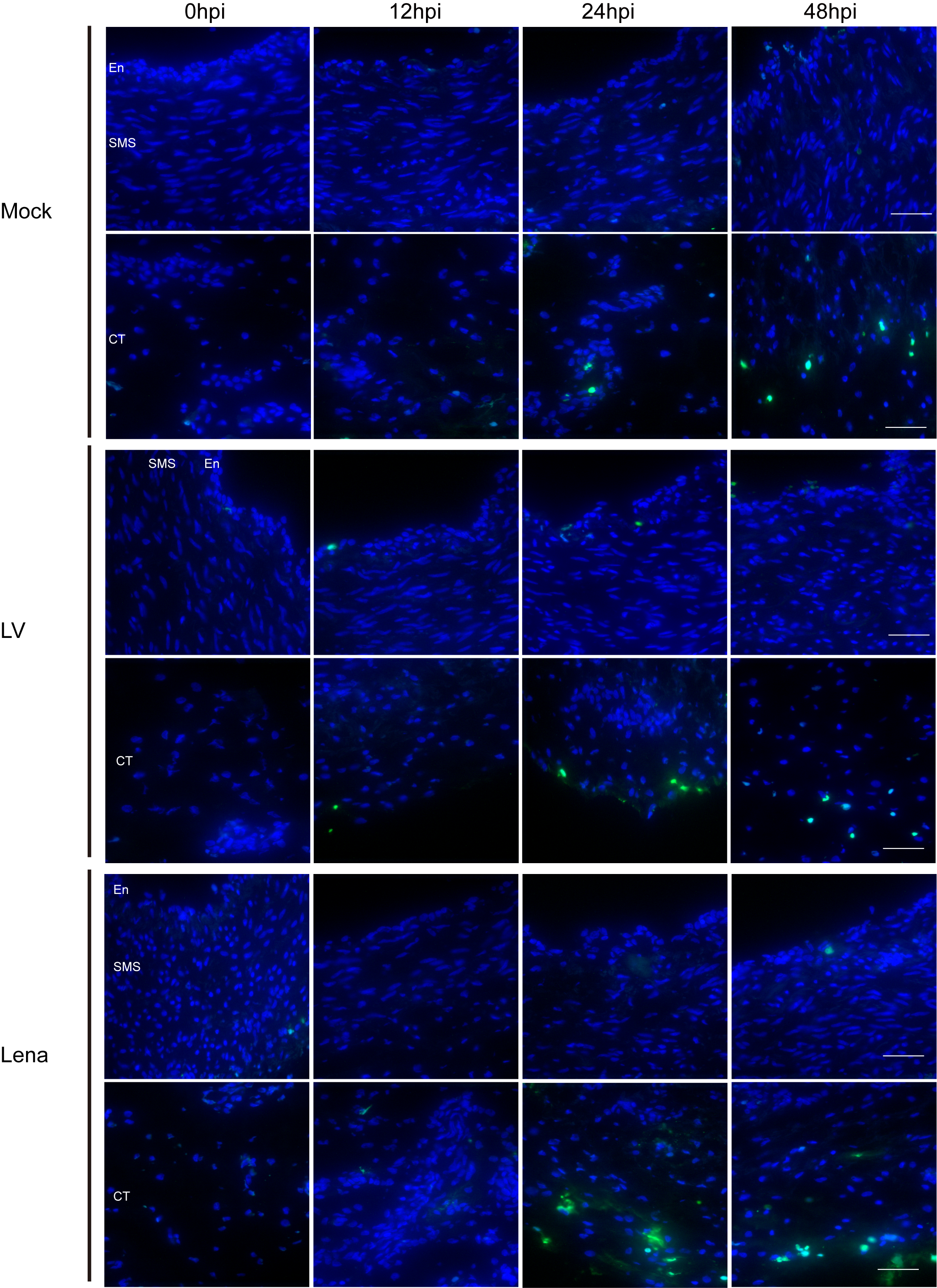

Supplement: Supplementary Figure 2 — Evaluation of cell viability in leg vein explants by TUNEL assay. TUNEL positive cells were quantitated in endothelial cell layers (En), smooth muscles cells (SMS) and connective tissues (CT) in the mock-inoculated group, LV-inoculated group and Lena-inoculated group at different time points. Scale bar: 50 μm. [file Image_2.tif]

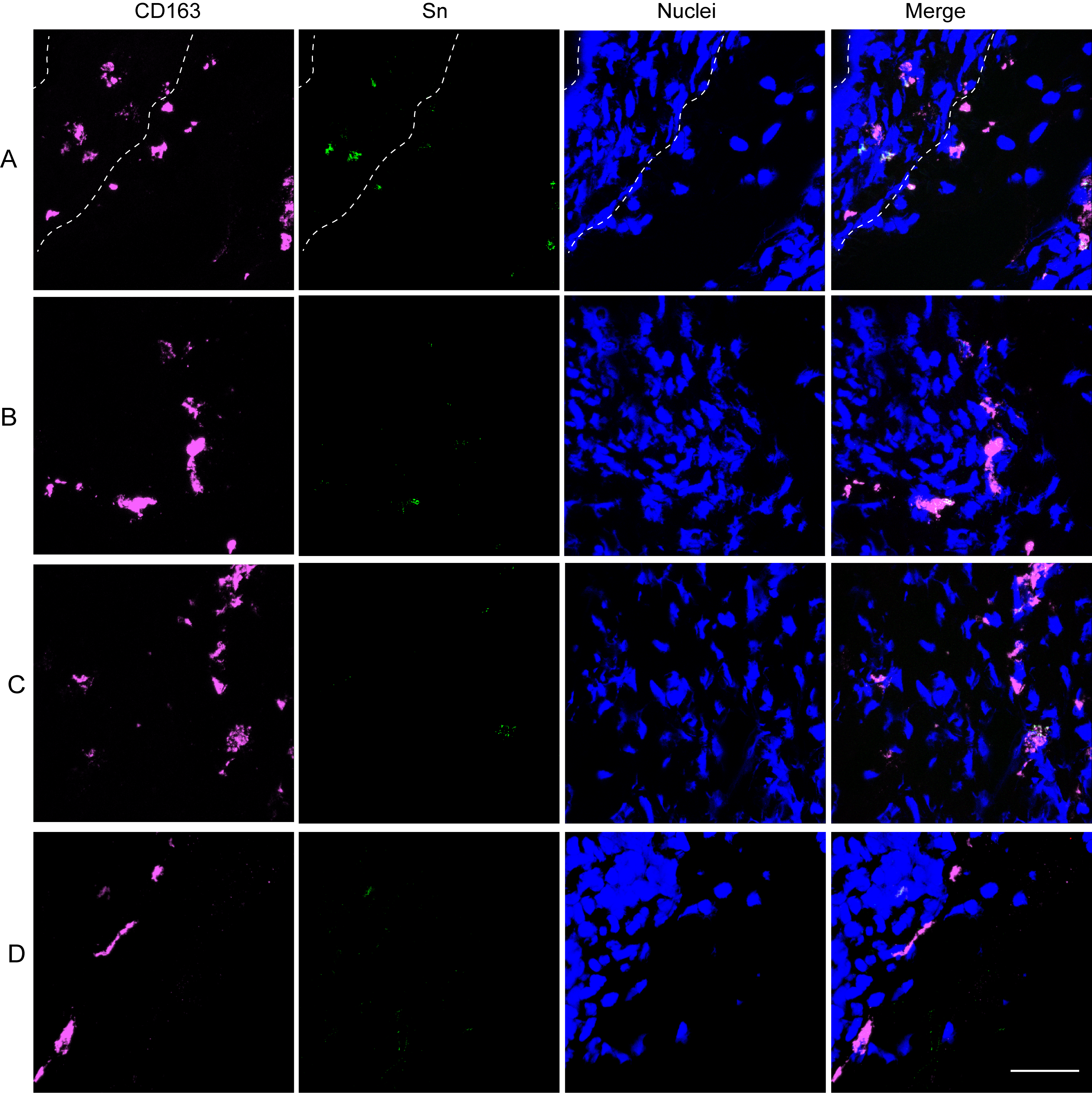

Supplement: Supplementary Figure 3 — Distribution of CD163+Sn+ and CD163+Sn- cells in different parts of ear vein explants. Cryosections of the vein explant were immunostained for CD163 (magenta) and Sn (green) with a double immunofluorescence staining. Nuclei were visualized with Hoechst (blue). For the quantitation and characterization of the perivenous macrophages in each ROI (area of 0.03 mm2 (30276 μm2)), a total number of around 100 cells in each ROI was analyzed. Scale bar: 50 μm. [file Image_3.tif]

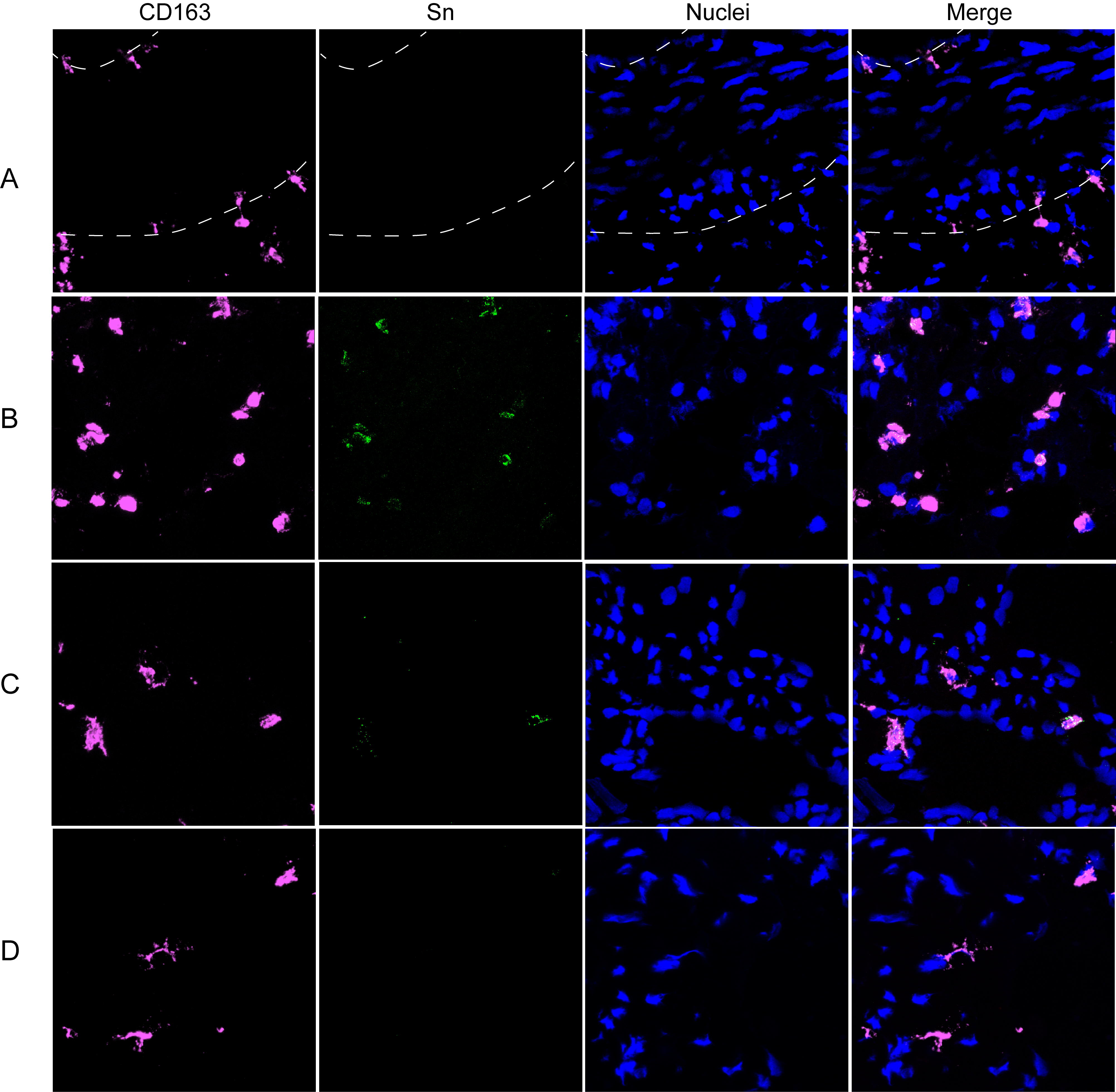

Supplement: Supplementary Figure 4 — Distribution of CD163+Sn+ and CD163+Sn- cells in different parts of leg vein explants. Cryosections of the vein explant were immunostained for CD163 (magenta) and Sn (green) with a double immunofluorescence staining. Nuclei were visualized with Hoechst (blue). For the quantitation and characterization of the perivenous macrophages in each ROI (area of 0.03 mm2 (30276 μm2)), a total number of around 100 cells in each ROI was analyzed. Scale bar: 50 μm. [file Image_4.tif]

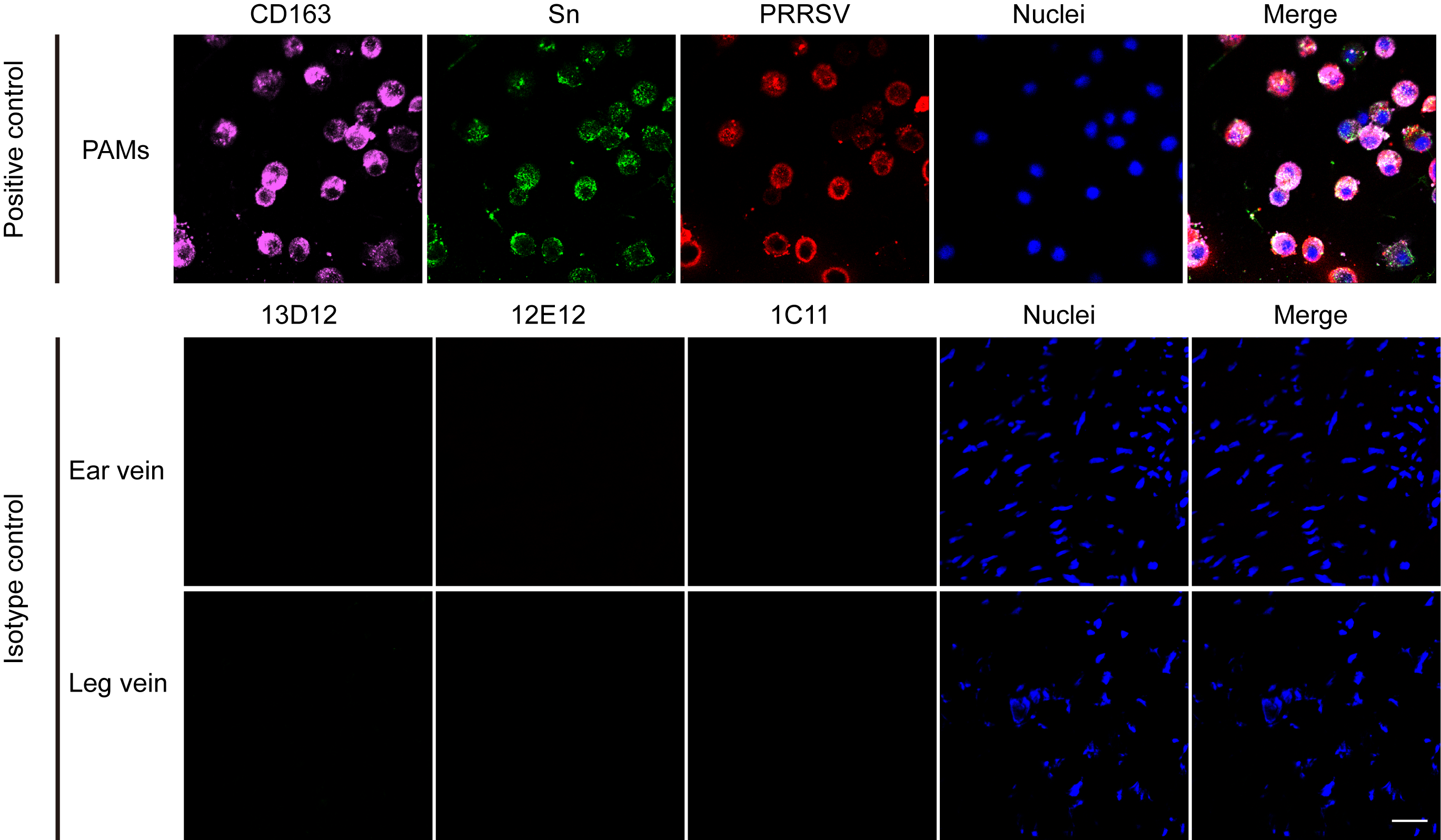

Supplement: Supplementary Figure 5 — Positive control and isotype controls for the CD163/Sn/PRRSV triple immunofluorescence staining. PAMs inoculated with PRRSV were used as positive control for the CD163/Sn/PRRSV triple immunofluorescence staining. A mouse anti-pseudorabies virus gD mAb, clone 13D12 (IgG1) (Nauwynck and Pensaert, 1995), a mouse anti-pseudorabies virus gB mAb, clone 1C11 (IgG2a) (Nauwynck and Pensaert, 1995) and a mouse anti-PCV2 capsid mAb, clone 12E12 (IgG2b) (Saha et al., 2012) were used as isotype controls for the ear and leg vein triple immunofluorescence staining. Scale bar: 25 μm. [file Image_5.tif]
